# Local topographic wetness indices predict household malaria risk better than land-use and land-cover in the western Kenya highlands

**DOI:** 10.1186/1475-2875-9-328

**Published:** 2010-11-16

**Authors:** Justin M Cohen, Kacey C Ernst, Kim A Lindblade, John M Vulule, Chandy C John, Mark L Wilson

**Affiliations:** 1 Department of Epidemiology, School of Public Health, University of Michigan, Ann Arbor, MI, USA; 2 National Center for Zoonotic, Vector-Borne, and Enteric Diseases, Centers for Disease Control and Prevention, Atlanta, GA, USA; 3 Kenya Medical Research Institute, Kisumu, Kenya; 4 Department of Pediatrics, University of Minnesota Medical School, Minneapolis, MN, USA

## Abstract

**Background:**

Identification of high-risk malaria foci can help enhance surveillance or control activities in regions where they are most needed. Associations between malaria risk and land-use/land-cover are well-recognized, but these environmental characteristics are closely interrelated with the land's topography (e.g., hills, valleys, elevation), which also influences malaria risk strongly. Parsing the individual contributions of land-cover/land-use variables to malaria risk requires examining these associations in the context of their topographic landscape. This study examined whether environmental factors like land-cover, land-use, and urban density improved malaria risk prediction based solely on the topographically-determined context, as measured by the topographic wetness index.

**Methods:**

The topographic wetness index, an estimate of predicted water accumulation in a defined area, was generated from a digital terrain model of the landscape surrounding households in two neighbouring western Kenyan highland communities. Variables determined to best encompass the variance in this topographic wetness surface were calculated at a household level. Land-cover/land-use information was extracted from a high-resolution satellite image using an object-based classification method. Topographic and land-cover variables were used individually and in combination to predict household-level malaria in the communities through an iterative split-sample model fitting and testing procedure. Models with only topographic variables were compared to those with additional predictive factors related to land-cover/land-use to investigate whether these environmental factors improved prediction of malaria based on the shape of the land alone.

**Results:**

Variables related to topographic wetness proved most useful in predicting the households of individuals contracting malaria in this region of rugged terrain. Other variables related to human modification of the environment also demonstrated clear associations with household malaria. However, these land-cover/land-use variables failed to produce unambiguous improvements in statistical predictive models controlling for important topographic factors, with none improving prediction of household-level malaria more than 75% of the time.

**Conclusions:**

Topographic wetness values in this region of highly varied terrain more accurately predicted houses at greater risk of malaria than did consideration of land-cover/land-use characteristics. As such, those planning control or local elimination strategies in similar highland regions may use topographic and geographic characteristics to effectively identify high-receptivity regions that may require enhanced vigilance.

## Introduction

Local elimination of malaria requires identification of residual transmission foci [1], while optimal targeting of interventions by control programs necessitates identifying areas of particularly high risk [2]. In either case, such foci are the results of heterogeneous *Plasmodium *transmission caused by unevenly distributed factors related to mosquito vectors, humans, and their interaction [3]. *Anopheles *vectors for malaria have species-specific environmental requirements for reproduction and development [4], as do the *Plasmodium *parasites that they transmit [5]. For these reasons, the environmental context in which people live is an important determinant of their degree of exposure to potentially infectious malaria vectors [2].

Patterns of disease are generally determined by vector and parasite ecologies [6] and, at finer spatial scales, topographic variations [7]. In addition, however, human interaction with local environmental contexts will modulate this baseline risk. Environmental modifications such as deforestation [8] or swamp reclamation [9] may alter malaria transmission dynamics through enhanced breeding site availability or increased temperature. In this way, agricultural changes may be responsible for increasing malaria transmission [10], whether by directly increasing standing water or indirectly via deforestation, both of which can increase ambient temperature and produce sunlit pools suitable for larval development. At the same time, such environmental change may produce increased community wealth, a more regular food supply, and thus decreased risk of disease [11]. Because of these complexities, considerable confounding may cloud the relationships between malaria risk and environmental factors, making it challenging to assess which of these determinants are most useful for predicting areas of high risk.

It was previously demonstrated that local incidence in an epidemic-prone region of the western Kenyan highlands manifested strong associations with topographically-determined wetness indices [7]. The topographic wetness index (TWI), an estimate of predicted water accumulation, succinctly describes the shape of the land at any given point on the landscape as the ratio of the uphill area from which water would flow into that point to the local slope at that point. In regions with varied terrain, these measures of topography, which can be easily calculated from freely-available digital elevation models and open-source geographic information system software packages, are highly interrelated with land-cover/land-use. For example, swampland in valley bottoms will have a high TWI, while high-altitude fields on hills used for tea cultivation typically will have a lower TWI. TWI may also at least partially determine the suitability of land for agriculture and pasture, both land-use types that have been found to be associated with larval habitat in the region [12-14], and thus by extension, malaria risk.

Because of these dependencies, the associations demonstrated between land-cover/land-use, TWI, and malaria risk may be distorted by unmeasured confounding. The utility of these measures for prediction of small-scale areas at high risk of malaria transmission, therefore, requires assessing these factors jointly. If wetness indices encompass the same information as land-cover/land-use, they may provide an easier alternative to costly and time-intensive satellite image classification for prediction of high-risk areas. The present investigation was designed to compare the accuracy of predictions based upon topography-related variables, including TWI and elevation, versus those based upon satellite-derived information on land-cover and land-use. In addition, it aimed to determine whether consideration of those variables related to land-cover, land-use, and urban density improved malaria risk prediction based solely on the topographic context in which they occurred.

## Methods

### Demographic, geographic, and epidemiologic data

As detailed in previous investigations of malaria at this study site [7,15], demographic and residence data were collected during 2003 and 2004 from all people living in the villages of Kapsisiywa and Kipsamoite in the western Kenyan highlands (> 1900 m), where the predominant vector found during entomologic evaluations was *An. gambiae *s.l. (97.5%) [15]. Latitude and longitude for each house was determined with a differential Trimble Pathfinder GPS (Trimble Navigation Ltd., Sunnyvale, CA) (resolution ± 1 m). Total person-years of occupancy for each household were calculated from residency information obtained through quarterly demographic censuses, providing a case-rate denominator for each household. Malaria events per household were calculated by summing the number of individuals from each household presenting with slide-confirmed *Plasmodium falciparum *malaria at the sole government clinics in Kipsamoite and Kapsisiywa. Individuals presenting with malaria more than once during the same year were counted as multiple cases if these events occurred >30 days apart. Ethical approval for the study was obtained from the Kenya Medical Research Institute National Ethical Review Committee and the Institutional Review Boards for Human Studies at the University of Michigan.

### Topographic variable data and sources

The topographic wetness index (TWI), an estimate of predicted water accumulation in a defined area, was calculated as the ratio of the area upslope (i.e., from where water would flow into that point) from any given point on the landscape to the local slope at that point, thus corresponding to the amount of water that should enter a given spatial unit divided by the rate at which the water should flow out of that unit. The TWI is an appealing measure because it represents a simple, biologically meaningful description of how topographic characteristics, like slope and surface curvature, may affect malaria risk via suitability for mosquito breeding. A 10 m resolution Digital Terrain Model (DTM) derived from topographic maps was obtained courtesy of BIOTA Subproject E02 [16]. In this DTM, elevation across the landscape was recorded in 10 m-by-10 m grid-cells. The DTM was georeferenced and verified for internal consistency against both a 90 m resolution digital elevation model created from Shuttle Radar Topography Mission (SRTM) satellite imagery and GPS ground readings obtained using a Trimble Pathfinder unit with 1 m accuracy. This georeferenced DTM was then inputted into the open-source GIS program SAGA [17] to produce the TWI layer for the 604,595 1-m^2 ^grid cells of the study area.

Characterizing the topography surrounding each house required finding discrete TWI variables that could encompass the continuous landscape of valleys, peaks, and slopes. Many candidate predictive variables derived from the TWI layer, including the local value; the minimum, maximum, average, and sum of wetnesses within various radii of houses; and the distance from houses to the nearest location in each of several wetness categories, were entered into a factor analysis. The individual wetness variables most correlated with each of the identified principal components were then identified.

In addition, three elevation variables also were calculated. First, the elevation of each household in the community was obtained from the 10-m resolution DTM. The same DTM then was used to calculate stream channels throughout the study site in SAGA using Tarboton's Deterministic Infinity flow algorithm [18]. The elevation of the stream channel closest to each house was computed as a second elevation variable. Finally, the vertical elevation of each household above the nearest stream channel was calculated as a relative measure of elevation above valley bottoms.

### Land-cover/land-use variables

An IKONOS satellite image of the study area (4-by-4 m color pixel, 1 m pan-sharpened), taken on 26 November, 2002 (the date closest to the study period of any image available for purchase), was orthorectified with PCI Geomatica 9 (PCI Geomatics, Inc., Richmond Hills, Ontario, Canada), using Toutin's model for satellite orbital modeling. Eleven ground control points (GCPs) of easily identified landscape characteristics (e.g. road intersections) were obtained with a differential GPS to orthorectify the image. The orthorectification process indicated a root-mean-square error of 1.34 m, comprised of 0.75 m in the x-direction and 1.12 m in the y-direction.

The object-based classification program eCognition Professional 4 (Definiens AG, München, Germany) was applied to the orthorectified image to derive variables related to human interaction with and modification of the local environment. The computer classification was checked by hand and misclassified features were corrected whenever errors were identified. Clouds and shadow covered 7% of the image, preventing complete classification of the land-cover surrounding certain houses. When ≥80% of land within 250 m of a house could not be classified, that house was excluded from analysis.

Proximity of households to environmental characteristics, such as natural and reclaimed swamp or roads, was calculated in ArcGIS 9.1 (ESRI, Redlands CA). The amount of farmland (tea and non-tea separately), pasture, and tree cover around houses was quantified. Measures were calculated separately for areas <50 m surrounding each house (representing the immediate environment in which people resided), and <500 m (more general context). Additionally, the number of neighbouring households within 50 m was calculated as a measure of local density. To encompass potential community-level differences not captured by the environmental and topographic variables, the community in which each household was located was considered as a binary variable.

### Data analysis

A negative binomial distribution was found to fit the number of cases per household better than a Poisson distribution, so negative binomial regression was used to examine associations between topographic and land-cover/land-use factors and household malaria. T-tests were used to compare houses with and without malaria according to the same variables. Additionally, this investigation aimed to identify factors that were not merely associated with household malaria, but also proved useful in predicting it (statistical association does not necessarily indicate useful predictive value [19] since relationships may be confounded by unmeasured variables). Thus, to ensure that those variables included in models demonstrated predictive ability whether or not they were statistically significant [20,21], the relationship between environmental variables and household malaria was assessed via a repeated split-sample procedure [22] whereby houses were randomly assigned to either training or validation datasets. Households located >1 km from the swampy lowlands bordering each community were excluded from analysis to limit the potentially confounding effects of socioeconomic variation, temperature, and other factors that might influence malaria risk on broader spatial scales.

For each of 300 repetitions, a random selection process was used to group ~75% of houses into the training set and the other 25% into the validation set. Only the houses in the training set were used to derive models to predict the number of malaria cases occurring in each household throughout 2003 and 2004. To do so, combinations of candidate predictive variables were entered into a series of regression models with a negative binomial outcome of the number of cases occurring in each household. The log of the number of person-years each house was occupied by residents during the study period was entered as an offset variable in every model to account for the fact that households with more residents (i.e. contributed person-time) were expected generally to have more malaria cases. Regression models accounting for potential spatial autocorrelation using spherical and exponential structures were also fit for comparison.

In each repetition, the accuracy of all of the fitted models was then tested in the validation set. Each fit model was used to predict the number of expected cases in each house. Predicted case-counts in the validation set were normalized in each repetition so that their sum was equal to the sum of all actual case-counts. The normalized predicted case-counts were compared against the actual case-counts by calculating mean-squared-error (MSE). The MSEs from a given repetition were compared to see which combination of variables produced the smallest MSE for that repetition. A random variable was used as a comparative benchmark: for each house, this variable's value was drawn at random from a normal distribution, and it was entered into each model to assess model performance with an explanatory factor known to be unrelated to either malaria or topography.

To examine whether human-modified environmental factors would improve prediction of models with only topographic variables, each of the land-cover/land-use factors was added in turn as an additional explanatory variable to the best-predicting topographic model from each repetition. In The best-predicting topographic model was selected for that repetition from among those models using only a combination of TWI or elevation variables. The models with and without the land-cover/land-use variables were compared to see whether any of these factors increased the predictive ability of the topographic variables alone. The percentage of the 300 repetitions in which the addition of each environmental variable improved model prediction was tallied.

## Results

Overall, 1,378 houses were occupied by about 7,700 residents during the two-year period. In Kipsamoite, 77 different individuals presented at the clinic with malaria from April through December 2003, while 120 were diagnosed during 2004. In Kapsisiywa, 246 different individuals had clinically-diagnosed malaria from April to December 2003, with 270 during 2004. A total of 144 of 710 (20.3%) Kipsamoite houses ever occupied during the study had at least one case, while 281 of 668 (42.1%) Kapsisiywa houses had at least one case. Of these houses, 1,318 (95.6%) were successfully georeferenced.

Four principal components associated with household malaria were extracted from the many candidate wetness variables. To limit the number of TWI variables used and maximize their interpretability, representative TWI variables that encompassed most of the variation from each component were selected. These included: 1) distance from each household to the very wettest points around the community and the maximum wetness within 1 km of each house, 2) the sum of all 10 m^2 ^wetness grid-cells between 500 m and 1 km of each house, 3) the proximity of houses to regions of very low predicted wetness, and 4) minimum wetness within 150 m. At the household level, these topographic variables were highly correlated with land-cover/land-use variables (Table 1).

**Table 1:** Correlation coefficients and their p-values for the associations between topographic and human-modified variables in Kipsamoite and Kapsisiywa.

	**Distance to very wet**	**Distance to very dry**	**Min. wetness** **within 150 m**	**Sum of wetness within 500 m to 1 km**	**Max. wetness** **within** **1 km**	**Elevation**
	
**Distance to reclaimed swamp**	0.35 < 0.0001	-0.19 < 0.0001	-0.40 < 0.0001	-0.40 < 0.0001	-0.40 < 0.0001	0.69 < 0.0001
	
**Distance to natural swamp**	0.79 < 0.0001	0.26 < 0.0001	-0.39 < 0.0001	-0.58 < 0.0001	-0.79 < 0.0001	0.49 < 0.0001
	
**Trees (500 m)**	-0.02 0.5053	-0.13 < 0.0001	-0.16 < 0.0001	-0.16 < 0.0001	-0.05 0.1090	0.09 0.0042
	
**Farmland (500 m)**	0.64 < 0.0001	0.01 0.6967	-0.53 < 0.0001	-0.67 < 0.0001	-0.68 < 0.0001	0.66 < 0.0001
	
**Tea (50 m)**	-0.13 < 0.0001	-0.01 0.6446	0.12 < 0.0001	0.14 < 0.0001	0.15 < 0.0001	-0.16 < 0.0001
	
**Pasture (500 m)**	0.09 0.0028	0.13 < 0.0001	0.09 0.0022	0.15 < 0.0001	-0.02 0.4692	-0.03 0.2781
	
**Houses (50 m)**	0.00 0.9997	0.07 0.0238	0.11 0.0002	0.09 0.0033	0.05 0.1215	-0.15 < 0.0001
	
**Distance to road**	-0.11 0.0004	0.07 0.0198	0.21 < 0.0001	0.14 < 0.0001	0.10 0.0006	-0.36 < 0.0001

### Associations with household malaria

As the number of neighbouring houses increased, the risk of disease in a given house declined. Over the entire study area, each additional neighbour within 25 m of a house was associated with an Incident Rate Ratio [IRR] = 0.73 (95% CI: 0.57-0.93), controlling for year, person-time, and community. The community of residence was strongly associated with malaria in the complete dataset, with Kapsisiywa houses having 2.63 (2.09-3.32) times the odds of malaria than Kipsamoite houses.

Houses with any malaria cases tended to be closer to the swamp (t = 7.17, 830 d.f., p < 0.001) and farther from roads (t = -2.81, 641 d.f., p = 0.005). Houses with malaria were surrounded by more trees (t = -2.76, 741 d.f., p = 0.006) and less farmland (t = 2.92, 765 d.f., p = 0.004). Distances to both natural swamp (for each 100 m increase in distance, IRR = 0.92 [0.91-0.94]) and reclaimed swamp (IRR = 0.86 [0.82-0.91]) were negatively associated with household malaria in the complete dataset. The amount of farmland within 500 m was significantly and negatively associated with malaria in the complete dataset (for a 10,000 m^2 ^increase, IRR = 0.94 [0.92-0.95]), while the amount of tea growing <500 m was positively associated with household malaria (IRR = 1.17 [1.11-1.24] for a 10,000 m^2 ^increase).

Each of the selected TWI variables was associated with household malaria. Houses located farther from the region's wettest points (IRR = 0.89 [0.87-0.91] per 100 m increase) or nearer to the driest points (IRR = 0.63 [0.51-0.76]) had lower average malaria case-counts. Houses with greater maximum wetness within 1 km (IRR = 1.42 [1.32-1.53] per 1-unit increase) or larger summed wetness indices within 500 m to 1 km (IRR = 1.02 [1.02-1.03] per 1000-unit increase) were also at higher risk. Finally, minimum wetness within 150 m also was positively associated with malaria (IRR = 1.43 [1.25-1.64]). Higher elevation was associated with reduced malaria risk (IRR = 0.86 [0.83-0.89] per 10 m increase). Accounting for spatial autocorrelation did not affect model results in any qualitative way.

### Predicting household malaria

**Figure 1:**
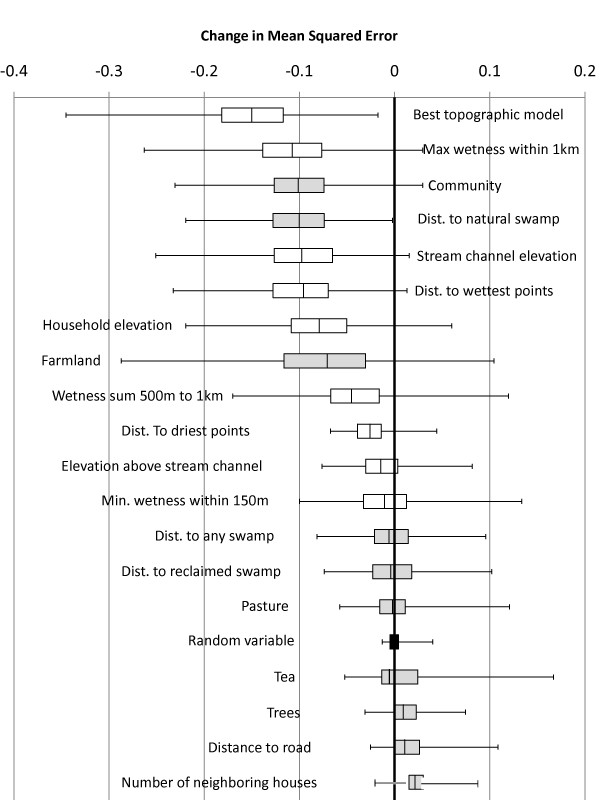
**Change in mean-squared-error resulting from the addition of topographic (white boxes) and land-cover/land-use (gray boxes) variables to predictive models with only a person-time offset**. The results of 300 split-sample repetitions are pictured (the box represents the 25^th ^to 75^th ^percentile, while whiskers show range). Negative values indicate that addition of the variable improved the accuracy of the model in predicting malaria risk; boxes crossing the zero line indicate prediction was improved in some random subsets of houses but not in others.

Models with only a person-time offset variable produced average MSE = 1.23. Variables related to both land-cover/land-use and TWI/elevation were found to improve predictive accuracy. Figure 1 depicts the distribution of changes in MSE for each variable from the 300 random samples of houses; the more negative the change in MSE, the greater the improvement in prediction caused by consideration of that variable. Of the land-cover/land-use variables, distance to swamplands, the community in which the household was located, and amount of farmland or pasture within 500 m each independently on average predicted malaria better than the random variable. The amount of farmland within 500 m improved 87% of person-time models, while the amount of tea <500 m, despite being positively associated with household malaria, improved the prediction of only 132 of 300 models (44%). In every model repetition, adding distance between houses and natural swampland improved prediction of malaria using person-time alone. However, distance to reclaimed swamp predicted malaria better than person-time alone in only half of the repetitions. Addition of the community variable (Kipsamoite or Kapsisiywa) variable to the models with person-time alone resulted in improved prediction 99% of the time.

Land-cover/land-use or elevation variables like maximum wetness <1 km, stream channel elevation, distance to wettest locations in the community, and household elevation improved prediction in nearly every case (Figure 1). The exact combination of these variables that comprised the best-fitting topographic model varied across the 300 repetitions, but the best-fitting model always improved prediction. The most common topographic variables included in the best-fitting model included distance to wettest locations, distance to driest locations, and elevation of the nearest stream channel.

**Figure 2:**
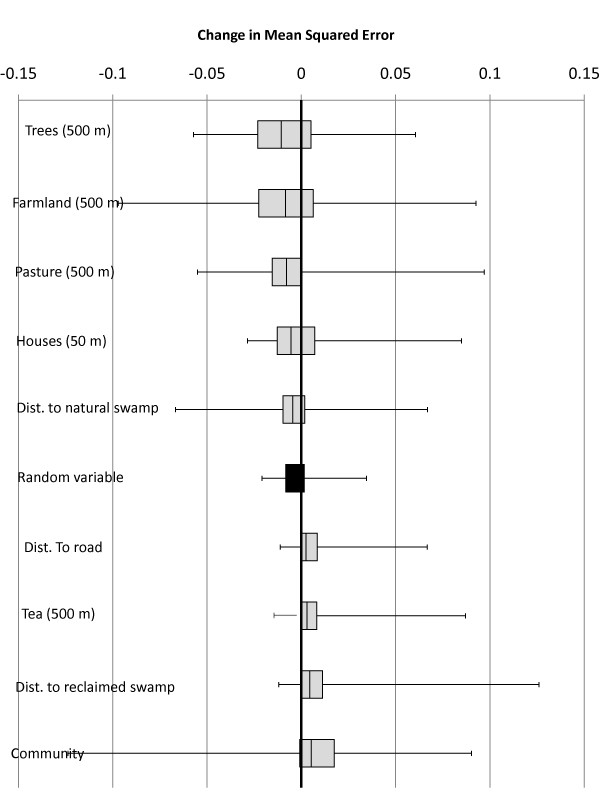
**Change in mean-squared-error resulting from the addition of land-cover/land-use variables to the best-fitting topographic predictive model for each random subset of houses**. The results of 300 split-sample repetitions are pictured (the box represents the 25^th ^to 75^th ^percentile, while whiskers show range). Negative values indicate that addition of the variable improved the accuracy of the model in predicting malaria risk.

None of the land-cover/land-use variables improved the prediction of these best-fitting topographic models more than 75% of the time (Figure 2). The amount of pasture within 500 m performed best, improving 225 of 300 repetitions (75%). Distances to natural swamp; the amount of pasture, farmland, and trees within 500 m; and the number of other houses within 50 m were the only variables that improved average prediction more than the random variable.

## Discussion

Accurate prediction of *Plasmodium *transmission risk is essential in heterogeneous environments to permit focal control measures and heightened surveillance in the regions that require them the most [23]. Such prediction requires simultaneous consideration of factors related to vector distribution, human-vector contact, human practices, and the environmental context in which they occur [3]. Results of this investigation corroborate previous studies demonstrating associations between malaria risk and both topographic characteristics, specifically TWI and elevation, and human-influenced environmental variables including land-cover and land-use. Importantly, however, they demonstrate that factors associated with malaria are not necessarily predictive of it, most likely because strong correlations between environmental factors can lead to confounded relationships.

The predictive models tested here indicate that land-cover/land-use variables failed to unambiguously improve prediction of household malaria risk based solely on topographic factors like the distance between a house and the highest TWI locations in the community, despite the fact that these variables were statistically associated with malaria risk. Given the cost and time required to classify land-cover/land-use data at high resolution, the superior accuracy of TWI in predicting high-risk foci suggests that it may be both a simpler and more useful remotely-sensed tool for risk prediction. These findings indicate that control programs operating in such rugged terrain may consider application of the TWI to derive risk maps in such settings. While the most predictive combination of topographic variables changed as different houses were randomly selected for model fitting, a subset of variables - including distance to the wettest and driest locations around the community and the distance to the nearest stream channel - were reliably included in a majority of these combinations, implying important commonalities.

Individual environmental variables, including distance to natural swamps, community of residence, and nearby farmland, each improved predictions of malaria risk over models considering person-time alone. However, none of the examined land-cover/land-use variables improved more than 75% upon the best-fitting topographic models (Figure 2), nor did combinations of variables perform better. In other words, little effect of land-cover/land-use was evident after accounting for general topographic patterns. Given that these human-modified environmental variables are strongly correlated with topographic variables (Table 1), they may be too highly interrelated to observe individual effects. Alternatively, the strong, overarching patterns of malaria risk in this specific region may be largely determined by the uneven topography and varied elevation, making it difficult to identify localized, nuancing effects of small-scale environmental modifications such as those examined here. It seems probable that repeating this analysis in a geographic setting with less topographic variation would produce different results. Thus, analyses similar to these in lowland regions with less extreme topographies are needed to evaluate whether these results are applicable elsewhere.

The community in which a house was located was, unsurprisingly, a good predictor of malaria risk since overall incidence was three times higher in Kapsisiywa than in Kipsamoite. However, after adding the best-predicting topographic variables (Figure 2), consideration of community rarely improved the models. This finding suggests that a great deal of the difference in malaria rates between these two neighbouring communities can be attributed to differences in their topographies.

Distance from households to swamp edges was a better predictor than distance to the stream channels that generally run through the center of swamps, corroborating studies of larval habitat that indicate swampy margins are more suitable than deeper waters for vector breeding [24]. However, distance to any swamp generally did not enhance prediction of malaria case-counts when added to models with the best-fitting topographic variables. This result indicates that the presence of "swampy" land-cover, marked by papyrus and other characteristic "natural" plants, as well as channeled cropland in its reclaimed form, was a less specific measure than the topographic variables describing the shape of the land.

More extensive agriculture surrounding houses, which primarily included cultivation of maize, beans, and other crops, was associated with reduced malaria risk. It is possible that crops decreased the suitability of land for mosquito breeding, either by channeling away standing water or by preventing direct sunlight from reaching water pooling under tall crops, but such an effect would run counter to the conclusions of other investigations [25]. Alternatively, large amounts of farmland may be indicative of greater SES, and the observed association accordingly may reflect such unmeasured variables [26]. These and other possibilities including specific crop types and cultivation methods should be more carefully evaluated.

Misclassification of environmental variables may have affected these results. Land-cover measures were derived from a single IKONOS image taken in 2002, and any changes in land-use or land-cover occurring after that time could have introduced error. Transects conducted at the study site in 2005 that georeferenced the location and type of fields, pastures, trees, and other land-cover/land-use matched closely with the classified satellite-image (data not shown), indicating that features had not changed greatly over the time period between the image and the study. Although farmers reported that crop types can change from month-to-month or from year-to-year, the location of fields remained stable over the study period. However, if human modification of the local environment causes only subtle changes in malaria risk from general topographic trends, even a small amount of error could compound the difficulty of observing such effects. Additionally, the TWI data described here was based on a single algorithm [27]; consideration of other methods for deriving TWI may yield different results.

Classification of satellite imagery is challenging, and methods for doing so have not improved greatly in recent years [28]. Elevation data, however, is easily processed into useful metrics like the topographic wetness indices computed here with the use of freely available GIS programs. The June 2009 release by NASA and Japan's Ministry of Economy, Trade, and Industry of a global 30 m digital elevation model derived from Advanced Spaceborne Thermal Emission and Reflection Radiometer (ASTER) imagery http://asterweb.jpl.nasa.gov/gdem.asp permits free access to global elevation data. These findings suggest that such data resources may be used in identifying other foci at high risk for malaria, at least in highly topographically variable regions. Although the IKONOS image used here was selected for its fine spatial resolution of 1 m^2^, replication of this analysis using other types of remotely-acquired imagery, such as Landsat or ASTER, would allow consideration of land-cover and land-use variables derived from alternative sources; the greater temporal resolution and use of different sensors would allow examination of other types of data that have previously been demonstrated to be important for malaria risk prediction [4,29].

The results of this study indicate that elevation and predicted accumulation of water are highly predictive of malaria patterns in this small region. People living in areas with a high TWI appeared to be at significantly greater risk of malaria than those living in areas of lower TWI. Other variables related to land-cover/land-use and human modification of the environment demonstrated associations with household malaria and improved prediction of models over that of person-time alone. However, these variables generally failed to produce unambiguous improvements in models controlling for important topographic factors, suggesting that their importance for determining patterns of malaria in this region was slight when accounting for the varied shape of the land around these communities. Future studies should assess the utility of TWI for malaria risk prediction across larger and more geographically diverse areas, especially at scales useful for Ministries of Health to target interventions; replication of this work in regions of both similarly rugged terrain and flatter, more arid areas will be required before conclusions can be drawn about the utility of these methods elsewhere. However, these results suggest that malaria control programs in similar highland regions might use topographic and geographic variance rather than land-cover/land-use to efficiently identify locations that are highly suitable for transmission and which may benefit from enhanced vigilance.

## Competing interests

The authors declare that they have no competing interests.

## Authors' contributions

JMC was responsible for conceptualization, data analysis and interpretation, drafting and revision of the manuscript. KCE and KAL were involved in data collection, interpretation of results, and critical revision of the manuscript. JMV provided institutional support for this study and reviewed the manuscript. CCJ was involved in data collection and interpretation of results and critical revision of the manuscript. MLW participated in conceptualization, analysis, interpretation and critical revision of the manuscript. All authors read and approved the final manuscript.
